# CXCL10 as a shared specific marker in rheumatoid arthritis and inflammatory bowel disease and a clue involved in the mechanism of intestinal flora in rheumatoid arthritis

**DOI:** 10.1038/s41598-023-36833-7

**Published:** 2023-06-16

**Authors:** Yin Guan, Yue Zhang, Yifan Zhu, Yue Wang

**Affiliations:** 1 Affiliated Hospital of Nanjing University of Chinese Medicine, Nanjing, 210029 Jiangsu China; 2 Department of Rheumatism Immunity Branch, Affiliated Hospital of Nanjing University of Chinese Medicine, No. 155 Hanzhong Road, Qinhuai, Nanjing, 210029 Jiangsu China

**Keywords:** Computational biology and bioinformatics, Rheumatology

## Abstract

This study aimed to identify shared specific genes associated with rheumatoid arthritis (RA) and inflammatory bowel disease (IBD) through bioinformatic analysis and to examine the role of the gut microbiome in RA. The data were extracted from the 3 RA and 1 IBD gene expression datasets and 1 RA gut microbiome metagenomic dataset. Weighted correlation network analysis (WGCNA) and machine learnings was performed to identify candidate genes associated with RA and IBD. Differential analysis and two different machine learning algorithms were used to investigate RA’s gut microbiome characteristics. Subsequently, the shared specific genes related to the gut microbiome in RA were identified, and an interaction network was constructed utilizing the gutMGene, STITCH, and STRING databases. We identified 15 candidates shared genes through a joint analysis of the WGCNA for RA and IBD. The candidate gene *CXCL10* was identified as the shared hub gene by the interaction network analysis of the corresponding WGCNA module gene to each disease, and *CXCL10* was further identified as the shared specific gene by two machine learning algorithms. Additionally, we identified 3 RA-associated characteristic intestinal flora (*Prevotella*, *Ruminococcus*, and *Ruminococcus bromii*) and built a network of interactions between the microbiomes, genes, and pathways. Finally, it was discovered that the gene *CXCL10* shared between IBD and RA was associated with the three gut microbiomes mentioned above. This study demonstrates the relationship between RA and IBD and provides a reference for research into the role of the gut microbiome in RA.

## Introduction

Rheumatoid arthritis (RA) is an autoimmune inflammatory condition that primarily affects the joints. Individuals at risk for and those with RA experience gut dysbiosis^1–3^. A small-sample study on patients with RA reported that most patients had subclinical intestinal inflammation^4^. Intestinal inflammation can be caused by and sustained through gut dysbiosis^5,6^. Inflammatory bowel disease (IBD), which encompasses Crohn’s disease (CD) and ulcerative colitis (UC), is a chronic, recurrent inflammatory illness of the gut with immune system disturbance^7^. Despite having different target organs, IBD and autoimmune rheumatic illnesses share a genetic foundation^8–10^.

According to a population-based study from South Korea, RA is strongly associated with IBD^11^. Patients with RA have aberrant intestinal barrier permeability, which is consistent with the intestinal alterations observed in patients with IBD^12^. The IL-23/IL-17 inflammatory axis is activated during the development of both RA and IBD^13^. The expression of *ENA78/CXCL5* is increased in tissues that are inflamed during RA and IBD^14,15^. Additionally, patients with RA and IBD have higher IL-6 levels^16–18^. These findings highlight the role of the gastrointestinal system in the development of RA and suggest a shared pathogenic mechanism between IBD and RA. However, the role of the gut microbiome in the pathogenesis of RA and the genetic interactions and molecular mechanisms underlying the relationship between RA and IBD remain unclear.

Machine learning technologies have been widely applied in the study of inflammatory diseases in recent years. Using machine learning and deep learning, Maria Giovanna Danieli et al. investigated how intravenous and subcutaneous immunoglobulin treatment affects patients with idiopathic inflammatory myopathies^19^. Isabelle Ayoub et al. used machine learning to assess the treatment response for lupus nephritis using standard clinical data with novel biomarkers^20^.

**Figure 1:**
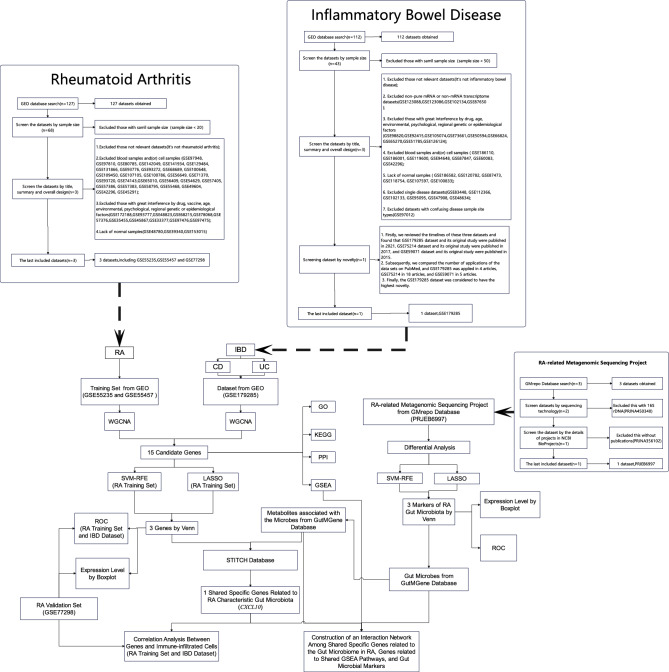
Flowchart of the analytical process.

In this study, bioinformatic tools were used to identify the common specific genes and processes between RA and IBD and examine the relationship between the gut microbiome in RA and the shared specific genes. We comprehensively analyzed four gene expression datasets from the Gene Expression Omnibus (GEO) database (GSE55235, GSE55457, GSE179285, and GSE77298) and an RA-related metagenomic sequencing dataset, PRJEB6997, from the GMrepo database. Weighted correlation network analysis (WGCNA) was performed to identify candidate genes associated with RA and IBD. Gene set enrichment analysis (GSEA) was performed to assess changes at the pathway level. The shared specific genes between RA and IBD were screened using two types of machine learning algorithms and receiver operating characteristic (ROC) curves. The characteristics of the gut microbiome in RA were examined using differential analysis, two types of machine learning algorithms, and ROC curves. Subsequently, the shared specific genes related to the gut microbiome in RA were identified, and an interaction network of these genes and those related to shared GSEA pathways were constructed using the gutMGene, STITCH, and STRING databases. The Spearman correlation analysis was used to determine the connection between these genes and immune cells. To the best of our knowledge, this study is the first to report the common genes associated with both RA and IBD and their relationship with the gut microbiome in RA using a systematic bioinformatic approach. Figure 1 demonstrates the study design.

## Materials and methods

### Search strategy for datasets

For RA, 127 datasets were systematically retrieved from the GEO database (https://www.ncbi.nlm.nih.gov/geo/) using the keywords: ((((rheumatoid arthritis[MeSH Terms]) OR rheumatoid arthritis) AND human[Organism]) AND Expression profiling by array[Filter]) AND (“2012/01/01” [Publication Date]: “2022/01/01” [Publication Date]). Exclusion criteria: (1) Excluded those with a small sample size (sample size < 20), (2) Excluded datasets that were irrelevant (it is not rheumatoid arthritis), (3) Excluded blood samples and/or cell samples, (4) Excluded samples with significant drug, vaccine, age, environmental, psychological, regional genetic, or epidemiological factors, and (5) Excluded samples lacking normal samples (listed in Fig. 1).

For IBD, 112 data sets were systematically retrieved from the GEO database (https://www.ncbi.nlm.nih.gov/geo/) using the keywords: ((((inflammatory bowel disease[MeSH Terms]) OR inflammatory bowel disease) AND human[Organism]) AND Expression profiling by array[Filter]) AND (“2012/01/01” [Publication Date]: “2022/01/01” [Publication Date]). Exclusion criteria: (1) Excluded those with a small sample size (sample size < 50), (2) Excluded datasets that were irrelevant (it is not inflammatory bowel disease), (3) Excluded impure mRNA or non-mRNA transcriptome datasets, (4) Excluded samples with significant drug, age, environmental, psychological, regional genetic, or epidemiological factors, (5) Excluded blood samples and/or cell samples, (6) Excluded samples lacking normal samples, (7) Excluded single disease datasets, and (8) Excluded datasets with ambiguous disease sample site types. Inclusion criteria (Novelty assessment): The one with the most recent publication date and the fewest studies on PubMed was selected based on the exclusion criteria (listed in Fig. 1).

For the intestinal flora of RA, three datasets were systematically retrieved from the GMrepo database^21^ (https://gmrepo.humangut.info/home) using the keywords: [Arthritis, Rheumatoid]. Exclusion criteria: (1) Excluded datasets with 16 s rDNA, (2) Excluded datasets without publications in NCBI BioProjects (listed in Fig. 1).

### Extraction and preprocessing of GEO data

The microarray datasets GSE55235^22^, GSE55457^22^, GSE179285^23^, and GSE77298^24^ were extracted from the GEO database (https://www.ncbi.nlm.nih.gov/geo/) using the R package GEOquery^25^. Additionally, *Homo sapiens* samples for the GSE55235 and GSE55457 datasets were generated using the GPL96 [HG-U133A] Affymetrix Human Genome U133A Array platform; those for the GSE179285 dataset were generated using the Agilent 014850 Whole Human Genome Microarray 4 × 44 K G4112F (Probe Name version) platform; and those for the GSE77298 dataset were generated using the GPL570 [HG-U133_Plus_2] Affymetrix Human Genome U133 Plus 2.0 Array platform. The GSE55235 dataset contained 10 synovial tissue samples from patients with RA and 10 samples from healthy individuals; the GSE55457 dataset contained 10 synovial tissue samples from healthy individuals and 13 samples from patients with RA; the GSE179285 dataset contained 14 inflamed colon tissue samples from patients with CD, 23 inflamed colon tissue samples from patients with UC, and 23 healthy colon tissue samples from healthy individuals; the GSE77298 dataset contained 16 synovial tissue samples from patients with RA and 7 samples from healthy individuals. The data from the GSE55235, GSE55457, and GSE77298 datasets were normalized using the RMA algorithm in the Affy package^26^ in R. The training set was created by merging the data from the GSE55235 and GSE55457 datasets, and the validation set was created using the data from the GSE77298 dataset. Additionally, the batch effects of the combined GSE55235 and GSE55457 datasets were removed using the Combat function of the sva package^27^. The principal component analysis (PCA) was used to evaluate the batch effect correction of the combined data^28^. Data from the GSE179285 dataset were downloaded, standardized, and analyzed using GEOquery^25^, Biobase^29^, and limma packages^30^. We used a distinct function of the “dplyr” package to remove the duplicate genes from each data set^31^. R software (version 4.3.0) was used for all data processing and analysis. Table 1 shows the comprehensive information for each dataset.

**Table 1:** Data information summary.

GEO accession	Platforms	Sample		Tissue (Homo sapiens)	Attribute	Author/reference
GSE55235	GPL96	Normal	10	Synovium	Training	Woetzel D^22^
		RA	10	Synovium		
GSE55457	GPL96	Normal	10	Synovium	Training	Woetzel D^22^
		RA	13	Synovium		
GSE179285	GPL6480	Normal	23	Uninflam colon		Keir ME^23^
		CD	14	Inflamed colon		
		UC	23	Inflamed colon		
GSE77298	GPL570	Normal	7	Synovium	Validation	Broeren MG^24^
		RA	16	Synovium		
BioProject accession		Sample		Tissue (microorganism)	Author/Reference	
PRJEB6997		Normal	55	Intestinal flora	Tisza MJ^79^	
		RA	92	Intestinal flora		

### Weighted gene co-expression network analysis

WGCNA is a standard method for processing large amounts of data that permits the grouping and modularization of a collection of genes most closely related to disease onset. The “WGCNA” R package was used in the study to build the gene co-expression network^32^. The gene expression matrix is first entered into the R software to check for missing data and identify outliers. Next, we developed a scale-free network to select a soft threshold value for each disease, which is used as the parameter cut-off value for creating the adjacency and topology matrices. Next, gene co-expression modules for each disease were identified using the block modules function and module division analysis. Each module was associated with these diseases (RA, CD, and UC), and the Pearson correlation coefficients were used to filter the most relevant modules. The genes in these modules were classified as genes associated with diseases. Finally, the Venn diagram package^33^ was used to overlap module genes associated with RA, CD, and UC to screen for shared candidate genes.The analysis images of WGCNA are generated by R software. R software is open source software, and the various packages are free and open source.

### Building a protein–protein interaction (PPI) network for module genes and identifying hub genes

The PPI network of module genes for each disease was investigated using the interaction relation in the database STRING (https://string-db.org/)^34^. The string result table was then entered into Cytoscape^35^. We performed Degree analysis to predict important nodes (or hub genes) using Cytoscape’s cytoHubba plugin^36^.

### Functional analysis and gene set enrichment analysis of candidate genes

The clusterProfiler package^37^ was used for GO and KEGG analyses of the candidate genes. Significant differences were defined as an adjusted P-value of ≤ 0.05. The STRING (https://string-db.org/)^34^ database was used to construct a PPI network of the candidate genes. Additionally, GSEA was performed using the clusterProfiler package to analyze genes associated with RA, CD, and UC (previously ranked based on their log2FC values between the analyzed groups). The “c2.cp.kegg.v7.5.1.symbols.gmt” gene set was used to identify significantly enriched genes with a nominal false discovery rate (FDR) of < 0.25 and P < 0.05.

### Screening and validation of the shared specific genes

LASSO^38^ was performed using the glmnet package^39^ to identify genes in the RA training set. In addition, the SVM-RFE algorithm^40^ in the e1071 package^41^ was used to select genes. A Venn diagram was created using the machine learning mentioned above to further identify key genes by overlapping genes in the two modules. Immediately, a boxplot demonstrating the expression of these genes in the RA training set, CD dataset, and RA validation set was created using the ggplot2^42^ and ggpubr^43^ packages. Furthermore, the function of these significant genes in the RA training set, CD dataset, UC dataset, and RA validation set was independently assessed by creating ROC curves in RStudio using the pROC package^44^. The shared specific genes in RA and IBD were selected as those with significant differences (P < 0.05) and AUC values of > 0.7 in all datasets.

### Identification of the gut microbiome in RA

The RA-related metagenomic sequencing dataset PRJEB6997^45^ was retrieved from the GMrepo database (https://gmrepo.humangut.info/home)^21^. A total of 147 samples, including 92 samples from patients with RA and 55 samples from healthy individuals, were selected from the PRJEB6997 database. The limma package in R was used for analyzing the differential abundance of gut microbes. Bacteria with significantly differential abundance were selected based on |log2 FC| values of > 3 and P < 0.05. The ggplot2 and ggrepel^46^ packages in R were used to create a volcano map to demonstrate bacteria with differential abundance. LASSO was performed using the glmnet package to identify potential specific bacteria. Additionally, the SVM-RFE algorithm in the e1071 package was used to select potential specific bacteria. Subsequently, a Venn diagram was developed to identify the markers of gut microbiota by overlapping bacteria in the two modules of LASSO and SVM-RFE. To examine the relationship between the gut microbial markers and RA, the pROC package was used to analyze ROC curves in RStudio, and the ggplot2 and ggpubr packages were used to construct a boxplot in RStudio to compare gene expression among groups.

### Identification of shared specific genes related to the gut microbiome in RA

The GutMGene (http://bio-annotation.cn/gutmgene/)^47^ database was used to identify metabolites from the markers of gut microbiota. The STITCH database (http://stitch.embl.de/)^48^ was used to identify shared specific genes directly associated with these metabolites.

### Construction of an interaction network among shared specific genes related to the gut microbiome in RA, genes related to shared GSEA pathways, and gut microbial markers

Venn diagrams were constructed to overlap pathways identified via GSEA in the RA training set, CD dataset, UC dataset, and RA validation set and to overlap genes associated with these pathways in the RA training set, CD dataset, UC dataset, and RA validation set. The relationship between the metabolites and markers of gut microbiota was analyzed using the gutMGene (http://bio-annotation.cn/gutmgene/) database. The STITCH database (http://stitch.embl.de/) was used to investigate the link between metabolites and genes(including genes associated with pathways identified via GSEA and the shared specific genes related to the gut microbiome in RA), and the STRING (https://string-db.org/)^34^ database was used for analyzing the interaction between genes associated with pathways identified via GSEA and the shared specific genes related to the gut microbiome in RA. Finally, the Cytoscape^35^ program was being used to combine and display the previously indicated network links, resulting in an interaction network of shared specific genes related to the gut microbiome in RA, genes associated with pathways identified via GSEA, and the markers of the gut microbiome.

### Correlation between shared specific genes associated with RA-specific gut microbiome and immune infiltration

We performed two immunoinvasive correlation analyses for this study. First, we combined the tagged genomes of several immune cell subpopulations in the CIBERSORT^49^, a deconvolution method to determine the proportion of 22 types of immune cells (reported in previous studies). In the meanwhile, we quantified the infiltration abundances of the 24 immune cells (reported in earlier studies) in these samples using ssGSEA^50^ based on the R package “GSVA”^51^. Finally, the association between immune cell infiltration in the RA training set, CD dataset, UC dataset, and RA validation set and shared specific genes connected to the gut microbiota in RA was analyzed using Spearman analysis.

## Results

### Screening of candidate genes associated with RA and IBD

**Figure 2:**
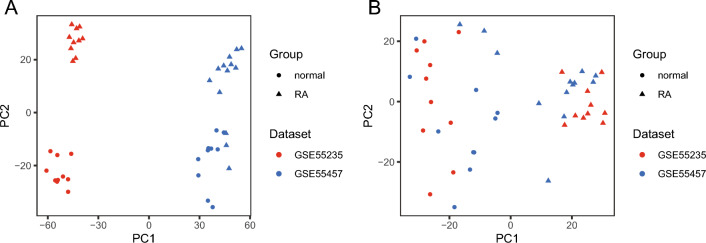
Principal component analysis (PCA) of combined data sets before and after batch effect removal. (**A**) PCA analysis was performed before batch effect elimination. (**B**) PCA analysis was performed after batch effect elimination. The GSE55235 dataset is red, and the GSE55457 dataset is blue. The triangular dots and the circular dots indicate samples from the RA and normal groups, respectively.

Before analysis, batch effect reduction was applied to the GSE55235 and GSE55457 data sets (the RA training set), and PCA was used to evaluate and compare the features. Before the batch effect was removed, the data were dispersed as data sets, and it was visible (Fig. 2A). The overall expression of the data was distributed in the form of sample treatment (normal and RA) and published more evenly than before after the batch effect was removed (Fig. 2B).

**Figure 3:**
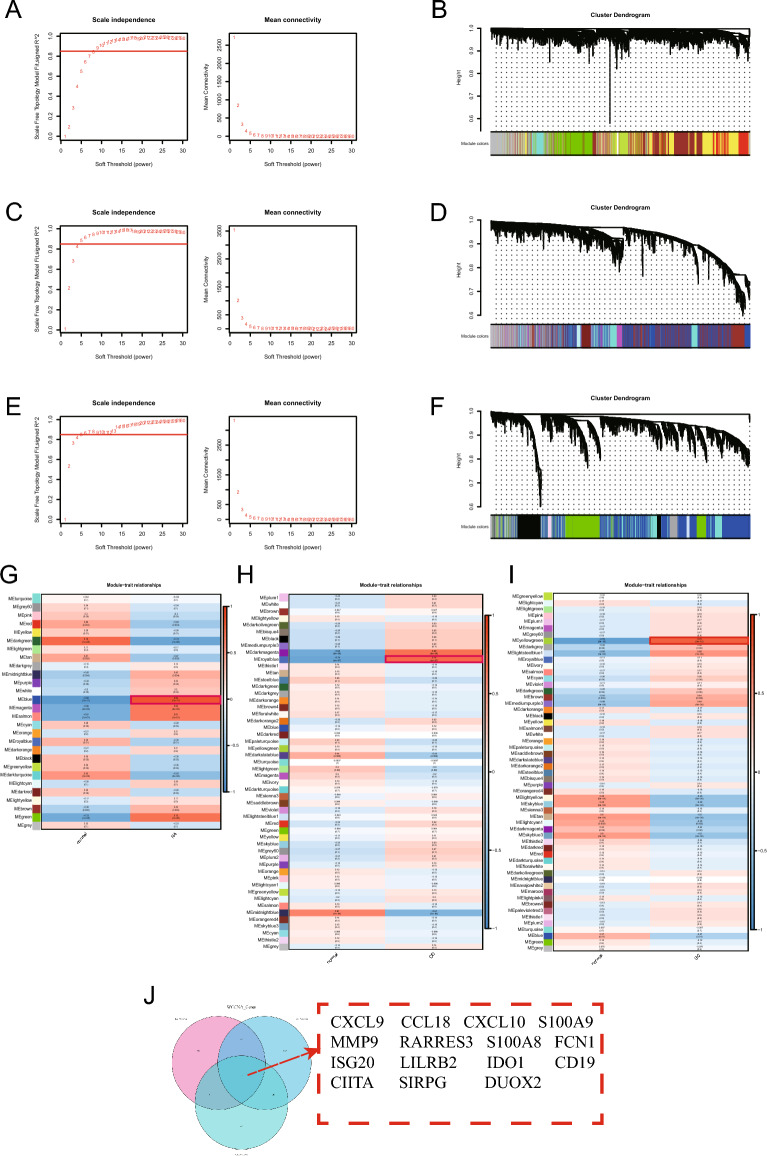
Potential genes implicated in both rheumatoid arthritis (RA) and inflammatory bowel disease (IBD) were discovered using WGCNA^52^. (**A**) Analysis of the network topology for RA utilizing various soft-threshold powers. (**B**) Determination of the gene modules that RA co-expresses. The 28 modules comprising the dendrogram’s branches are each assigned a different color. (**C**) A study of network topology for Crohn’s disease (CD) utilizing various soft-threshold powers. (**D**) Determination of the gene modules that CD co-expresses. The 52 modules comprising the dendrogram’s branches are each assigned a different color. (**E**) Analysis of the network topology for ulcerative colitis (UC) utilizing various soft-threshold powers. (**F**) Identification of the gene modules co-expressed by UC. The 57 modules comprising the dendrogram’s branches are each assigned a different color. (**G**) Heatmap depicting the association between the prevalence of RA and module genes. (**H**) Heatmap depicting the association between the prevalence of CD and module genes. (**I**) Heatmap depicting the association between the prevalence of UC and module genes. Red and blue show a positive and negative association, respectively, with the hue’s depth indicating each’s strength. (**J**) Venn diagram demonstrating the overlap between candidate genes of two IBD (CD and UC) modules and those of one RA module.

To construct a RA scale-free network, the soft threshold (R^2^ = 0.85) was set to 9 (Fig. 3A). To construct a CD scale-free network, the soft threshold (R^2^ = 0.85) was set to 5 (Fig. 3C). To construct a UC scale-free network, the soft threshold (R^2^ = 0.85) was set to 6 (Fig. 3E). Finally, the WGCNA revealed 28 gene modules associated with the occurrence of RA in the RA training set (Fig. 3B). Each module was identified using a different color. Genes in the “blue” module had a significant positive association with RA (blue module: r = 0.82, P = 1e − 11; Fig. 3G; Supplementary Tables S1, S2, Supplementary Spreadsheet S3). The “royal blue” module was among the 52 modules identified in the CD dataset (Fig. 3D) that had a significant positive association with CD (royal blue module: r = 0.74, P = 4e − 07; Fig. 3H; Supplementary Tables S4, S5, Supplementary Spreadsheet S6). The “yellow-green” module was among the 57 modules identified in the UC dataset (Fig. 3F) that had a significant positive association with UC (yellow-green module: r = 0.78, P = 3e − 10; Fig. 3I; Supplementary Tables S7, S8, Supplementary Spreadsheet S9). A total of 15 candidate genes associated with both RA and IBD were identified after the intersection of genes in the abovementioned target modules: *CXCL9, CCL18, CXCL10, S100A9, MMP9, RARRES3, S100A8, FCN1, ISG20, LILRB2, IDO1, CD19, CIITA, SIRPG,* and *DUOX2* (Fig. 3J).

### Construction of the PPI network of module genes and identification of hub genes

**Figure 4:**
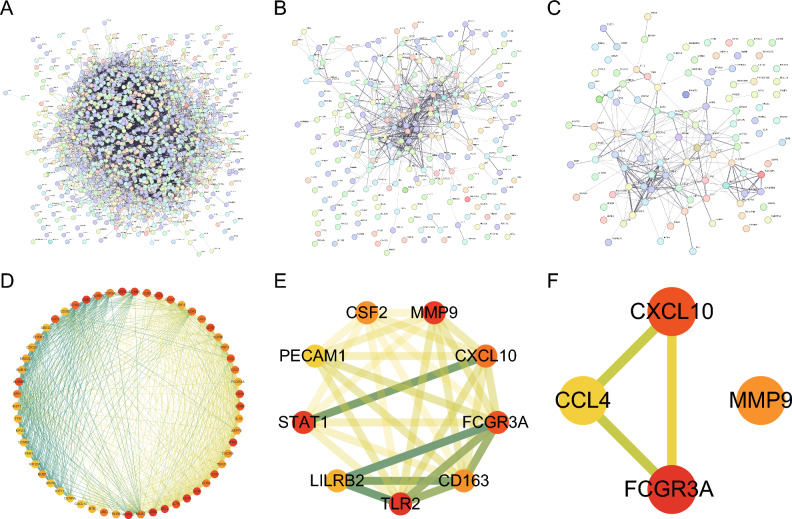
Protein–protein interaction network analysis of the corresponding WGCNA module genes for rheumatoid disease (RA) and inflammatory bowel disease (IBD) (Crohn’s disease [CD] and ulcerative colitis [UC]). (**A**) Protein interaction network of RA’s corresponding WGCNA module genes. (**B**) Protein interaction network of CD’s corresponding WGCNA module genes. (**C**) Protein interaction network of UC’s corresponding WGCNA module genes. (**D**) Network diagram of the hub nodes from RA. (**E**) Network diagram of the hub nodes from CD. (**F**) Network diagram of the hub nodes from UC.

We entered 1266 module genes from the RA’s corresponding WGCNA module (Fig. 4A), 239 module genes from the CD’s corresponding WGCNA module (Fig. 4B), and 111 module genes from the UC’s corresponding WGCNA module (Fig. 4C) into the STRING database to visualize the PPI network to investigate further whether the 15 candidate shared genes are the hub genes of the corresponding WGCNA module of each disease. Subsequently, the string result table was entered into Cytoscape. The cytoHubba tool was used to search hub nodes in the network, and the top 5% of genes were selected as hub gene nodes using Degree. The score increases as the node color darkens, and the number of edge interactions increases as the line color increases. The hub genes of the WGCNA module corresponding to RA include *CD19* and *CXCL10* (Fig. 4D; Supplementary Spreadsheet S10). The hub genes of the WGCNA module corresponding to CD include *CXCL10*, *LILRB2*, and *MMP9* (Fig. 4E; Supplementary Spreadsheet S11). The hub genes of the WGCNA module corresponding to UC include *CXCL10* and *MMP9* (Fig. 4F; Supplementary Spreadsheet S12).

### Functional annotation of candidate genes and identification of pathways associated with RA and IBD

**Figure 5:**
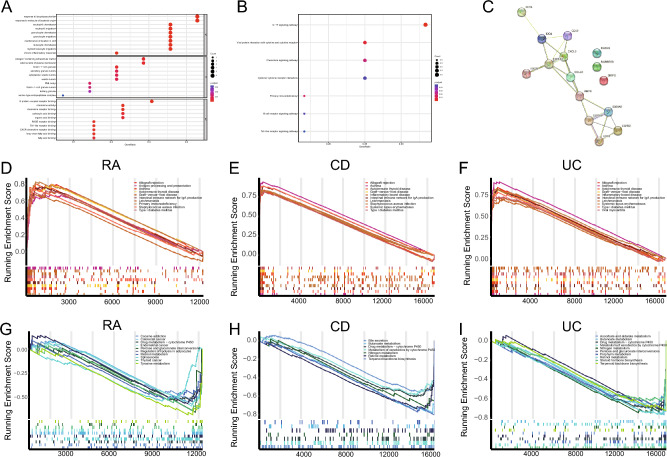
Functional annotation of candidate genes and identification of pathways associated with RA and IBD. (**A**) Results of GO enrichment analysis of 15 candidate genes identified via WGCNA. (**B**) Results of KEGG enrichment analysis^53^ of 15 candidate genes identified via WGCNA. (**C**) PPI network of 15 candidate genes. (**D**) Upregulated enriched pathways identified via GSEA in the RA training cohort. (**E**) Upregulated enriched pathways identified via GSEA in the CD cohort. (**F**) Upregulated enriched pathways identified via GSEA in the UC cohort. (**G**) Downregulated enriched pathways identified via GSEA in the RA training cohort. (**H**) Downregulated enriched pathways identified via GSEA in the CD cohort. (**I**) Downregulated enriched pathways identified via GSEA in the UC cohort.

The abovementioned 15 candidate genes were subjected to GO (Table 2) and KEGG (Table 3) functional enrichment analyses. The results of GO analysis revealed that the candidate genes were primarily associated with neutrophil chemotaxis, collagen-containing extracellular matrix, and chemokine activity (Fig. 5A). The results of KEGG analysis revealed the candidate genes were primarily associated with the IL-17 signaling pathway, chemokine signaling pathway, and cytokine–cytokine receptor interaction (Fig. 5B). Subsequently, the STRING database was used to construct a PPI network to visualize the interaction among the 15 candidate genes (Fig. 5C). The pathways associated with these genes in the RA training (Table 4), CD (Table 5) and UC (Table 6) cohorts were identified via GSEA. In the RA training cohort, pathways related to the intestinal immune network for IgA production, allograft rejection, and antigen processing and presentation were activated, whereas those related to retinol metabolism, regulation of lipolysis in adipocytes, and tyrosine metabolism were inhibited (Fig. 5D,G). In the CD cohort, pathways related to IBD, the intestinal immune network for IgA production, and asthma were activated, whereas those related to the metabolism of xenobiotics by cytochrome P450, metabolism of drugs by cytochrome P450, and butanoate metabolism were inhibited (Fig. 5E,H). In the UC cohort, pathways related to IBD, asthma, and the intestinal immune network for IgA production were activated, whereas those related to the metabolism of xenobiotics by cytochrome P450 and metabolism of drugs by cytochrome P450 were inhibited (Fig. 5F,I).

**Table 2:** GO enrichment summary.

ONTOLOGY	ID	Description	p.adjust	Count
BP	GO:0030593	Neutrophil chemotaxis	< 0.001	5
BP	GO:1990266	Neutrophil migration	< 0.001	5
BP	GO:0071621	Granulocyte chemotaxis	< 0.001	5
BP	GO:0097530	Granulocyte migration	< 0.001	5
BP	GO:0032496	Response to lipopolysaccharide	< 0.001	6
BP	GO:0002237	Response to molecule of bacterial origin	< 0.001	6
BP	GO:0002544	Chronic inflammatory response	< 0.001	3
BP	GO:0051651	Maintenance of location in cell	< 0.001	5
BP	GO:0030595	Leukocyte chemotaxis	< 0.001	5
BP	GO:0097529	Myeloid leukocyte migration	< 0.001	5
CC	GO:0062023	collagen-Containing extracellular matrix	0.003	4
CC	GO:0009897	external side of plasma membrane	0.003	4
CC	GO:0101002	Ficolin-1-rich granule	0.003	3
CC	GO:0034774	Secretory granule lumen	0.008	3
CC	GO:0060205	Cytoplasmic vesicle lumen	0.008	3
CC	GO:0031983	Vesicle lumen	0.008	3
CC	GO:0016605	PML body	0.011	2
CC	GO:1904813	Ficolin-1-rich granule lumen	0.014	2
CC	GO:0070820	Tertiary granule	0.021	2
CC	GO:1905370	Serine-type endopeptidase complex	0.034	1
MF	GO:0008009	Chemokine activity	< 0.001	3
MF	GO:0042379	Chemokine receptor binding	< 0.001	3
MF	GO:0050786	RAGE receptor binding	< 0.001	2
MF	GO:0035325	Toll-like receptor binding	< 0.001	2
MF	GO:0001664	G protein-coupled receptor binding	< 0.001	4
MF	GO:0045236	CXCR chemokine receptor binding	< 0.001	2
MF	GO:0036041	Long-chain fatty acid binding	< 0.001	2
MF	GO:0031406	Carboxylic acid binding	0.003	3
MF	GO:0043177	Organic acid binding	0.004	3
MF	GO:0005504	Fatty acid binding	0.004	2

**Table 3:** KEGG enrichment summary.

ID	Description	p.adjust	qvalue	Count
hsa04657	IL-17 signaling pathway	< 0.001	< 0.001	4
hsa04061	Viral protein interaction with cytokine and cytokine receptor	0.006	0.005	3
hsa05340	Primary immunodeficiency	0.016	0.012	2
hsa04062	Chemokine signaling pathway	0.021	0.015	3
hsa04662	B cell receptor signaling pathway	0.045	0.034	2
hsa04060	Cytokine-cytokine receptor interaction	0.046	0.034	3
hsa04620	Toll-like receptor signaling pathway	0.049	0.036	2

**Table 4:** RA GSEA enrichment summary.

ID	Description	Enrichment Score	pvalue	qvalue	Core_enrichment
hsa05340	Primary immunodeficiency	0.831	< 0.001	< 0.001	BLNK/IL7R/CD3D/ PTPRC/IL2RG/RFX5/ CD79A/TAP1/LCK/ CD8A/CD19/CD3E/ BTK/CD4/CIITA/ CD40/ZAP70
hsa05310	Asthma	0.808	< 0.001	< 0.001	HLA-DRB4/ HLA-DMB/ HLA-DMA/ HLA-DPB1/ HLA-DRA/HLA-DOB/ HLA-DQB1/ HLA-DRB1/ HLA-DPA1/ HLA-DQA1/ FCER1G/CD40
hsa05330	Allograft rejection	0.803	< 0.001	< 0.001	HLA-DRB4/HLA-DMB/ HLA-DMA/ HLA-DPB1/ HLA-DRA/HLA-DOB/ GZMB/HLA-DQB1/ HLA-DRB1/ HLA-DPA1/ HLA-DQA1/PRF1/ FAS/HLA-F/CD86/ CD40/CD28/HLA-B/ HLA-DOA/HLA-C/ HLA-E/FASLG/ HLA-G/CD40LG
hsa05320	Autoimmune thyroid disease	0.798	< 0.001	< 0.001	HLA-DRB4/ HLA-DMB/ HLA-DMA/ HLA-DPB1/ HLA-DRA/HLA-DOB/ GZMB/HLA-DQB1/ HLA-DRB1/ HLA-DPA1/ HLA-DQA1/PRF1/ FAS/HLA-F/ CD86/CD40/CD28/ HLA-B/HLA-DOA/ HLA-C/HLA-E/ FASLG/HLA-G/ CTLA4/CD40LG
hsa04672	Intestinal immune network for IgA production	0.769	< 0.001	< 0.001	TNFRSF17/ HLA-DRB4/ HLA-DMB/ HLA-DMA/ HLA-DPB1/CXCL12/ HLA-DRA/HLA-DOB/ CXCR4/HLA-DQB1/ HLA-DRB1/ HLA-DPA1/ HLA-DQA1/IL15/ IL15RA/ITGA4/ CD86/CD40/ TNFSF13/ITGB7
hsa05150	Staphylococcus aureus infection	0.745	< 0.001	< 0.001	HLA-DRB4/FCGR1A/ HLA-DMB/FCGR2B/ HLA-DMA/ITGB2/ HLA-DPB1/ HLA-DRA/ HLA-DOB/CFB/ HLA-DQB1/ HLA-DRB1/ HLA-DPA1/ HLA-DQA1/ ITGAM/FCGR3A/ SELPLG/FCGR2A/ C3AR1/FPR3/ C1QA/FCGR2C/ FCGR3B/C2/ITGAL
hsa04940	Type I diabetes mellitus	0.725	< 0.001	< 0.001	HLA-DRB4/ HLA-DMB/ HLA-DMA/ HLA-DPB1/ HLA-DRA/HLA-DOB/ GZMB/HLA-DQB1/ HLA-DRB1/ HLA-DPA1/ HLA-DQA1/ PRF1/FAS/ HLA-F/CD86/ CD28/HLA-B/ HLA-DOA/HLA-C/ HLA-E
hsa05332	Graft-versus-host disease	0.713	< 0.001	< 0.001	HLA-DRB4/HLA-DMB/ HLA-DMA/HLA-DPB1/ HLA-DRA/HLA-DOB/ GZMB/HLA-DQB1/ HLA-DRB1/HLA-DPA1/ HLA-DQA1/PRF1/ FAS/HLA-F/ CD86/CD28/ HLA-B/HLA-DOA/ HLA-C/HLA-E/ KLRC1/FASLG/ HLA-G
hsa05140	Leishmaniasis	0.684	< 0.001	< 0.001	NCF1/HLA-DRB4/ FCGR1A/HLA-DMB/ HLA-DMA/ITGB2/ STAT1/CYBB/ HLA-DPB1/ HLA-DRA/ HLA-DOB/PRKCB/ HLA-DQB1/PTPN6/ HLA-DRB1/ HLA-DPA1/ HLA-DQA1/ITGAM/ FCGR3A/FCGR2A/ CYBA/MARCKSL1/ FCGR2C/NCF4/ NCF2/FCGR3B/ MAPK13/ITGA4/ JAK2
hsa04612	Antigen processing and presentation	0.679	< 0.001	< 0.001	HLA-DRB4/ HLA-DMB/ HLA-DMA/ HLA-DPB1/ HLA-DRA/HLA-DOB/ RFX5/TAP1/ HLA-DQB1/TAPBP/ CD8A/CD74/ HLA-DRB1/ HLA-DPA1/ HLA-DQA1/IFI30/ CTSS/CD4/ HLA-F/CIITA/ CTSB/PSME1/ CALR/HLA-B/ HLA-DOA/HLA-C/ HLA-E/KLRC1/ LGMN/CD8B/ HLA-G
hsa00830	Retinol metabolism	− 0.525	0.008	0.024	RDH16/RETSAT/ CYP1A2/CYP3A4/ ALDH1A2/CYP2B6/ ALDH1A1/CYP2W1/ ALDH1A3/DHRS3/ ADH5/AOX1/ CYP26B1/ADH1C/ ADH1B
hsa05030	Cocaine addiction	− 0.529	0.007	0.023	CREB5/ATF4/ SLC6A3/JUN/ MAOA/FOSB
hsa00982	Drug metabolism—cytochrome P450	− 0.535	0.004	0.016	GSTM5/CYP2B6/ GSTM3/GSTT2/ GSTA4/GSTM1/ GSTM2/ADH5/ HPGDS/AOX1/ ADH1C/FMO2/ MAOA/ADH1B
hsa03040	Spliceosome	− 0.54	< 0.001	< 0.001	SF3B3/U2AF2/ TXNL4A/HNRNPM/ NCBP2/U2AF1/ ACIN1/THOC2/ MAGOH/SRSF8/ SF3A1/SNW1/ SNRPD1/TCERG1/ FUS/SNRNP40/ SNRNP70/XAB2/ PRPF31/SRSF10/ PUF60/SF3A3/ PRPF40A/DDX5/ SNRPA1/HSPA6/ SNRPB/CDC5L/ SF3B2/WBP11/ SRSF3/DHX38/ SRSF7/SNRPE/ PRPF3/BCAS2/ HNRNPA1/PQBP1/ EIF4A3/SRSF4/ SRSF6/HNRNPK/ SRSF5/SF3B1/ PRPF6/HNRNPU/ TRA2B/HSPA1A/ RBM25
hsa04923	Regulation of lipolysis in adipocytes	− 0.568	< 0.001	0.004	ADCY3/IRS1/ NPR1/NPY1R/ AQP7/AKT2/ LIPE/ABHD5/ PIK3R1/CIDEC/ PNPLA2/ADCY2/ IRS2/PLIN1/ PTGS2/FABP4
hsa05210	Colorectal cancer	− 0.575	< 0.001	< 0.001	TGFB2/CTNNB1/ GADD45G/AKT2/ SMAD3/AREG/ PIK3R1/TCF7L1/ TCF7L2/EREG/ TGFBR2/PMAIP1/ JUN/GADD45A/ CDKN1A/MYC/ EGFR/FOS/ GADD45B
hsa05213	Endometrial cancer	− 0.592	< 0.001	0.001	PTEN/CTNNB1/ GADD45G/AKT2/ PIK3R1/TCF7L1/ TCF7L2/FOXO3/ GADD45A/CDKN1A/ MYC/EGFR/ GADD45B
hsa05216	Thyroid cancer	− 0.642	< 0.001	0.002	CTNNB1/GADD45G/ TCF7L1/PPARG/ TCF7L2/GADD45A/ CDKN1A/MYC/ GADD45B
hsa00040	Pentose and glucuronate interconversions	− 0.655	0.008	0.024	UGP2/DCXR/ UGDH/AKR1B1/ AKR1B10
hsa00350	Tyrosine metabolism	− 0.683	< 0.001	0.002	AOC2/PNMT/ ADH5/AOX1/ ADH1C/AOC3/ FAH/MAOA/ ADH1B

**Table 5:** CD GSEA enrichment summary.

ID	Description	Enrichmentscore	pvalue	qvalue	Core_enrichment
hsa05310	Asthma	0.943	< 0.001	< 0.001	HLA-DRA/HLA-DRB3/HLA-DRB1/HLA-DRB5/ HLA-DRB4/HLA-DPB1/HLA-DPA1/ HLA-DQB1/HLA-DMA/ CCL11/HLA-DMB/HLA-DQA1/HLA-DOA/FCER1G
hsa05322	Systemic lupus erythematosus	0.891	< 0.001	< 0.001	HLA-DRA/HLA-DRB3/ HLA-DRB1/HLA-DRB5/ C1QB/FCGR3A/C1QA/ HLA-DRB4/HLA-DPB1/ HLA-DPA1/HLA-DQB1/ HLA-DMA/HLA-DMB/ C2/C1QC/HLA-DQA1/ HLA-DOA/CD86
hsa04672	Intestinal immune network for IgA production	0.89	< 0.001	< 0.001	HLA-DRA/HLA-DRB3/ HLA-DRB1/HLA-DRB5/ HLA-DRB4/MADCAM1/ HLA-DPB1/HLA-DPA1/ HLA-DQB1/HLA-DMA/ HLA-DMB/PIGR/HLADQA1/ IL15RA/TNFSF13B/ HLA-DOA/CCL28/CD86
hsa05150	Staphylococcus aureus infection	0.841	< 0.001	< 0.001	HLA-DRA/HLA-DRB3/ HLA-DRB1/HLA-DRB5/ C1QB/DEFA6/FCGR3A/ C1QA/DEFA5/HLA-DRB4/ HLA-DPB1/HLA-DPA1/ HLA-DQB1/HLA-DMA/ HLA-DMB/C2/FPR1/ C1QC/HLA-DQA1/C3AR1/ HLA-DOA
hsa05330	Allograft rejection	0.838	< 0.001	0.001	HLA-DRA/HLA-DRB3/ HLA-DRB1/HLA-DRB5/ HLA-DRB4/HLA-DPB1/ HLA-DPA1/HLA-DQB1/ HLA-DMA/GZMB/ HLA-DMB/HLA-DQA1/ HLA-DOA/HLA-F/ CD86/FAS
hsa05321	Inflammatory bowel disease	0.834	< 0.001	< 0.001	HLA-DRA/HLA-DRB3/ HLA-DRB1/HLA-DRB5/ HLA-DRB4/HLA-DPB1/ HLA-DPA1/HLA-DQB1/ HLA-DMA/HLA-DMB/ STAT1/HLA-DQA1/ TLR5/TLR2/HLA-DOA
hsa05140	Leishmaniasis	0.83	< 0.001	< 0.001	HLA-DRA/HLA-DRB3/ HLA-DRB1/NOS2/ HLA-DRB5/FCGR3A/ HLA-DRB4/HLA-DPB1/ HLA-DPA1/HLA-DQB1/ HLA-DMA/HLA-DMB/ STAT1/NCF2/HLA-DQA1/ TLR2/HLA-DOA
hsa05320	Autoimmune thyroid disease	0.825	< 0.001	< 0.001	HLA-DRA/HLA-DRB3/ HLA-DRB1/HLA-DRB5/ HLA-DRB4/HLA-DPB1/ HLA-DPA1/HLA-DQB1/ HLA-DMA/GZMB/ HLA-DMB/IFNA5/ HLA-DQA1/HLA-DOA/ HLA-F/CD86/TG/ TSHB/FAS/IFNA6
hsa05332	Graft-versus-host disease	0.822	< 0.001	0.002	HLA-DRA/HLA-DRB3/ HLA-DRB1/HLA-DRB5/ HLA-DRB4/HLA-DPB1/ HLA-DPA1/HLA-DQB1/ HLA-DMA/GZMB/ HLA-DMB/HLA-DQA1/ HLA-DOA/HLA-F/CD86/FAS
hsa04940	Type I diabetes mellitus	0.815	< 0.001	0.002	HLA-DRA/HLA-DRB3/ HLA-DRB1/HLA-DRB5/ HLA-DRB4/HLA-DPB1/ HLA-DPA1/HLA-DQB1/ HLA-DMA/GZMB/ HLA-DMB/HLA-DQA1/ HLA-DOA/HLA-F/CD86/FAS
hsa04976	Bile secretion	− 0.565	0.002	0.014	SLC9A3/RXRA/ NR1H4/ATP1A3/ UGT2B10/UGT2B7/ AQP1/SLC4A2/ ABCB1/UGT2B15/UGT1A6/ ADCY6/UGT2B17/ CFTR/AQP8
hsa00980	Metabolism of xenobiotics by cytochrome P450	− 0.586	< 0.001	0.009	CYP2A6/AKR7A3/ALDH3B/ DHDH/GSTA1/GSTP1/ GSTA3/CYP1A2/UGT2B10/ AKR7A2/UGT2B7/UGT2B15/ UGT1A6/CYP2B6/CYP2S1/UGT2B17/ADH1C/ADH1A
hsa00982	Drug metabolism—cytochrome P450	− 0.606	0.001	0.012	CYP2A6/ALDH3B1/GSTA1/ GSTP1/GSTA3/CYP1A2/ UGT2B10/UGT2B7/UGT2B1/ UGT1A6/MAOA/CYP2B6/ UGT2B17/ADH1C/ADH1A
hsa00910	Nitrogen metabolism	− 0.729	0.003	0.026	CA12/CA9/CA1
hsa00830	Retinol metabolism	− 0.74	< 0.001	< 0.001	CYP2A6/ALDH1A2/DGAT1/ CYP1A2/UGT2B10/UGT2B7/ DHRS4/RETSAT/RDH11/ UGT2B15/RDH5/UGT1A6/ DHRS9/CYP2B6/CYP2S1/ UGT2B17/ADH1C/ADH1A
hsa00650	Butanoate metabolism	− 0.781	< 0.001	0.001	HMGCL/ACADS/ BDH1/HMGCS2
hsa00900	Terpenoid backbone biosynthesis	− 0.804	< 0.001	0.002	PMVK/FNTB/ MVD/HMGCS2

**Table 6:** UC GSEA enrichment summary.

ID	Description	EnrichmentScore	pvalue	qvalue	Core_enrichment
hsa05310	Asthma	0.894	< 0.001	< 0.001	HLA-DRA/HLA-DRB5/ HLA-DRB1/HLA-DRB3/ HLA-DRB4/HLA-DPB1/ CCL11/HLA-DQB1/ HLA-DMA/HLA-DQA1/ HLA-DPA1/HLA-DMB/PRG2
hsa05330	Allograft rejection	0.829	< 0.001	< 0.001	HLA-DRA/HLA-DRB5/ HLA-DRB1/HLA-DRB3/ HLA-DRB4/HLA-DPB1/ CD86/HLA-DQB1/HLA-DMA/ HLA-DQA1/HLA-DPA1/ GZMB/HLA-DMB/FAS
hsa05322	Systemic lupus erythematosus	0.816	< 0.001	< 0.001	HLA-DRA/HLA-DRB5/ HLA-DRB1/HLA-DRB3/C1QB/ HLA-DRB4/HLA-DPB1/CD86/ FCGR3A/C1QA/HLA-DQB1/ HLA-DMA/HLA-DQA1/ HLA-DPA1/C2/HLA-DMB
hsa04672	Intestinal immune network for IgA production	0.809	< 0.001	< 0.001	HLA-DRA/HLA-DRB5/HLA-DRB1/ MADCAM1/HLA-DRB3/ HLA-DRB4/HLA-DPB1/CD86/ HLA-DQB1/HLA-DMA/ HLA-DQA1/HLA-DPA1/ TNFSF13B/HLA-DMB/IL15RA
hsa05320	Autoimmune thyroid disease	0.806	< 0.001	< 0.001	HLA-DRA/HLA-DRB5/ HLA-DRB1/HLA-DRB3/ HLA-DRB4/HLA-DPB1/CD86/ HLA-DQB1/HLA-DMA/ HLA-DQA1/HLA-DPA1/TPO/ TG/GZMB/HLA-DMB/FAS
hsa05332	Graft-versus-host disease	0.802	< 0.001	< 0.001	HLA-DRA/HLA-DRB5/ HLA-DRB1/HLA-DRB3/ HLA-DRB4/HLA-DPB1/CD86/ HLA-DQB1/HLA-DMA/ HLA-DQA1/HLA-DPA1/GZMB/ HLA-DMB/FAS
hsa04940	Type I diabetes mellitus	0.775	< 0.001	< 0.001	HLA-DRA/HLA-DRB5/ HLA-DRB1/HLA-DRB3/ HLA-DRB4/HLA-DPB1/CD86/ HLA-DQB1/HLA-DMA/ HLA-DQA1/HLA-DPA1/GZMB/ HLA-DMB/FAS
hsa05416	Viral myocarditis	0.72	< 0.001	< 0.001	HLA-DRA/HLA-DRB5/ HLA-DRB1/HLA-DRB3/ HLA-DRB4/HLA-DPB1/CD86/ HLA-DQB1/HLA-DMA/ HLA-DQA1/HLA-DPA1/ HLA-DMB/ITGB2
hsa05321	Inflammatory bowel disease	0.706	< 0.001	0.002	HLA-DRA/HLA-DRB5/ HLA-DRB1/HLA-DRB3/ HLA-DRB4/HLA-DPB1/ HLA-DQB1/HLA-DMA/ HLA-DQA1/HLA-DPA1/ IL21/HLA-DMB
hsa05140	Leishmaniasis	0.703	< 0.001	< 0.001	HLA-DRA/NOS2/ HLA-DRB5/HLA-DRB1/ HLA-DRB3/HLA-DRB4/ HLA-DPB1/FCGR3A/ HLA-DQB1/HLA-DMA/ HLA-DQA1/HLA-DPA1/ HLA-DMB/ITGB2/ PTPN6/STAT1/NCF4/ NCF2/CR1L/MAPK13/ TAB2/MAPK14
hsa00140	Steroid hormone biosynthesis	− 0.624	0.005	0.033	CYP3A4/SULT2B1/CYP21A2/ CYP3A5/HSD3B1/UGT2B10/ DHRS11/UGT1A6/HSD17B2/ CYP1A2/UGT2B7/UGT2B15/ HSD11B2/UGT2B17
hsa00980	Metabolism of xenobiotics by cytochrome P450	− 0.66	< 0.001	0.002	ALDH3B1/CYP3A5/CYP2A13/ AKR7A3/GSTA3/GSTA5/ GSTP1/MGST1/UGT2B10/ DHDH/UGT1A6/CYP1A2/ UGT2B7/UGT2B15/CYP2B6/ ADH1C/UGT2B17/ADH1A
hsa00860	Porphyrin metabolism	− 0.691	0.002	0.017	COX10/UROD/UGT2B10/UGT1A6/ UGT2B7/UGT2B15/UGT2B17
hsa00982	Drug metabolism—cytochrome P450	− 0.711	< 0.001	< 0.001	GSTA3/GSTA5/GSTP1/ MGST1/UGT2B10/UGT1A6/ CYP1A2/UGT2B7/UGT2B15/ MAOA/CYP2B6/ADH1C/ UGT2B17/ADH1A
hsa00040	Pentose and glucuronate interconversions	− 0.714	0.002	0.019	UGT2B10/AKR1B10/DHDH/ UGT1A6/UGT2B7/UGT2B15/ UGT2B17
hsa00053	Ascorbate and aldarate metabolism	− 0.72	0.002	0.019	UGT1A8/ALDH7A1/ALDH3A2/ GUSB/MIOX/UGT2B10/UGT1A6/ UGT2B7/UGT2B15/UGT2B17
hsa00900	Terpenoid backbone biosynthesis	− 0.724	0.002	0.017	MVD/FNTB/HMGCS2
hsa00910	Nitrogen metabolism	− 0.748	0.004	0.028	CA5A/CPS1/GLUD2/ CA9/CA7/CA12/CA4/CA1
hsa00830	Retinol metabolism	− 0.764	< 0.001	< 0.001	DHRS4/UGT2B10/DHRS9/ RETSAT/UGT1A6/CYP1A2/ RDH5/UGT2B7/RDH11/ UGT2B15/CYP2B6/ADH1C/ UGT2B17/ADH1A
hsa00650	Butanoate metabolism	− 0.767	< 0.001	0.004	BDH1/ACADS/HMGCS2

### Machine learning algorithm-based screening and validation of shared specific genes

**Figure 6:**
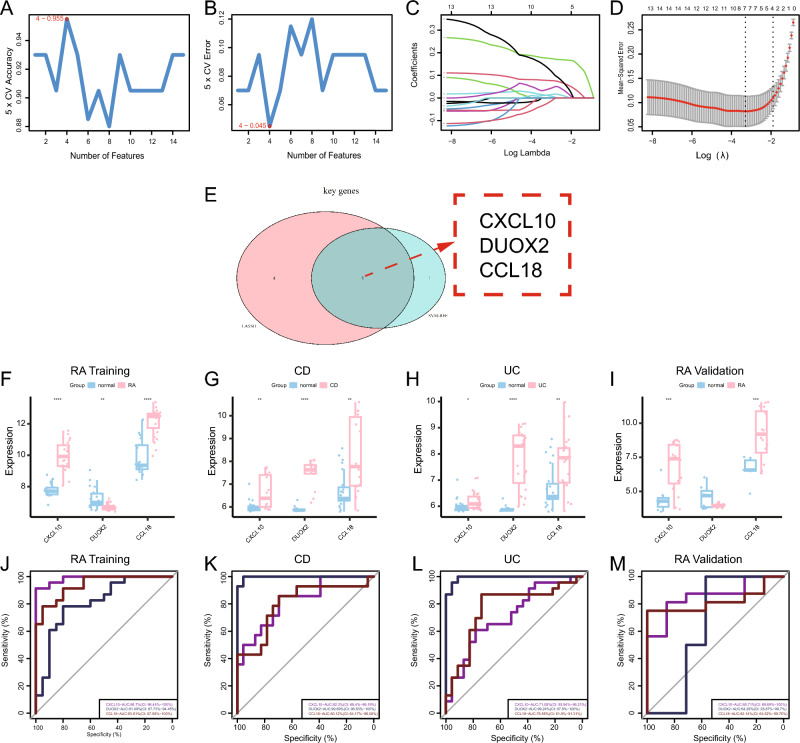
Machine learning-based identification and validation of potential shared specific genes. (**A**,**B**) Four genes were identified using the SVM-RFE algorithm in the RA training set. (**C**) LASSO coefficient profiles of 15 candidate genes in the RA training set. (**D**) LASSO coefficient profiles of 7 genes were selected as optimal (lambda) in the RA training set. (**E**) Venn diagram depicting the three key genes related to IBD in the RA training set. (**F**) Expression of *CXCL10, DUOX2,* and *CCL18* in the RA training set. (**G**) Expression of *CXCL10, DUOX2,* and *CCL18* in the CD dataset. (**H**) Expression of *CXCL10, DUOX2,* and *CCL18* in the UC dataset. (**I**) Expression of *CXCL10, DUOX2,* and *CCL18* in the RA validation set. (**J**) ROC curve for the verification of discriminative efficiency in the RA training set. (**K**) ROC curve for the verification of discriminative efficiency in the CD dataset. (**L**) ROC curve for the verification of discriminative efficiency in the UC dataset. (**M**) ROC curve for the verification of discriminative efficiency in the RA validation set (****P < 0.0001; ***P < 0.001; **P < 0.01; *P < 0.05).

The RA training set was selected to screen for key genes between RA and IBD using two different machine learning algorithms. Of the 15 candidate genes, 4 were identified using the SVM-RFE algorithm (Fig. 6A,B), and 7 were identified using the LASSO regression algorithm (Fig. 6C,D). Eventually, three key genes (*CXCL10, DUOX2,* and *CCL18*) that were commonly identified using these two algorithms were selected (Fig. 6E). We performed differential expression and ROC curve discriminative efficacy demonstration to determine whether these three key genes are shared specific genes of RA and IBD. In the RA training set, *CXCL10* and *CCL18* had a high expression difference, while *DUOX2* had a low expression difference (Fig. 6F); *CXCL10, DUOX2,* and *CCL18* expressed fair discriminative efficiency (AUC > 0.7) (Fig. 6J). In the CD dataset, *CXCL10, DUOX2,* and *CCL18* had high expression difference (Fig. 6G); *CXCL10, DUOX2,* and *CCL18* expressed fair discriminative efficiency (AUC > 0.7) (Fig. 6K). In the UC dataset, *CXCL10, DUOX2,* and *CCL18* had high expression difference (Fig. 6H); *CXCL10, DUOX2,* and *CCL18* expressed fair discriminative efficiency (AUC > 0.7) (Fig. 6L). In the RA validation set, *CXCL10* and *CCL18* showed high expression difference, whereas *DUOX2* showed no significant difference (Fig. 6I); *CXCL10* and *CCL18* expressed fair diagnostic efficiency (AUC > 0.7), whereas *DUOX2* expressed poor discriminative efficiency (AUC < 0.7) (Fig. 6M). The shared specific genes in RA and IBD were selected as those with significant differences (P < 0.05) and AUC values of > 0.7 in all datasets for further analysis (*CXCL10* and *CCL18*).

### Identification of the gut microbiome in RA based on machine learning

**Figure 7:**
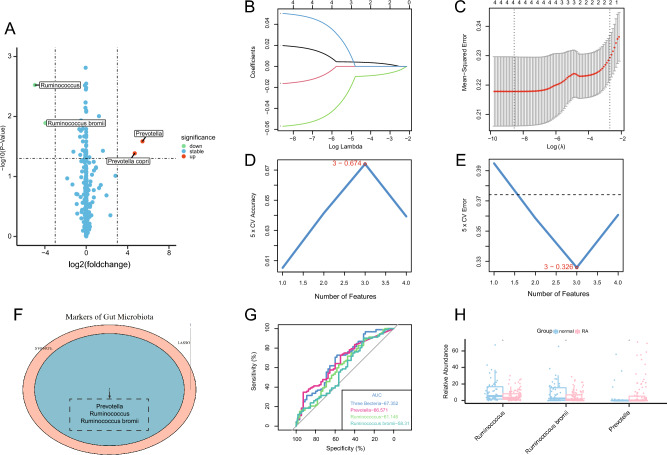
Identification of gut microbes associated with RA. (**A**) Volcano map demonstrating the differential abundance of intestinal microbes based on the criteria of |log2 FC| values of > 3 and P < 0.05. (**B**) LASSO coefficients of four intestinal microbes in the PRJEB6997 dataset. (**C**) LASSO coefficients of four microbes selected as optimal (lambda) in the PRJEB6997 dataset. (**D**,**E**) The PRJEB6997 dataset was screened using the SVM-RFE algorithm to identify three diagnostic indicators. (**F**) Venn diagram demonstrating the three ideal diagnostic biomarkers in the PRJEB6997 dataset. (**G**) ROC curve for the verification of diagnostic efficiency in the PRJEB6997 dataset. (**H**) Relative abundance of three bacterial groups (*Prevotella*, *Ruminococcus*, and *Ruminococcus bromii*) in the PRJEB6997 dataset (*P < 0.05).

Differential analysis revealed two intestinal microbes at the genus (*Prevotella* and *Ruminococcus*) and two intestinal microbes at the species (*Prevotella copri* and *Ruminococcus bromii*) levels in the PRJEB6997 dataset (Fig. 7A; Supplementary Spreadsheet S13). Gut microbes associated with RA were screened using two machine learning algorithms in the PRJEB6997 dataset. The LASSO regression algorithm revealed four microbial groups associated with RA (Fig. 7B,C), whereas the SVM-RFE algorithm revealed three microbial groups (Fig. 7D,E). The three overlapping microbial groups (*Prevotella*, *Ruminococcus*, and *Ruminococcus bromii*) identified using the two methods were selected (Fig. 7F), and their diagnostic efficacy and abundance were examined. *Prevotella*, *Ruminococcus*, and *Ruminococcus bromii* exhibited lower diagnostic values (0.5 < AUC < 0.7) (Fig. 7G). The abundance of these three bacterial groups was different between healthy and RA. The abundance of *Prevotella* was high and that of *Ruminococcus* and *Ruminococcus bromii* was low among patients with RA (Fig. 7H).

### Construction of an interaction network among shared specific gene associated with the gut microbiome in RA, genes related to shared pathways identified via GSEA, and RA-specific gut microbiome

**Figure 8:**
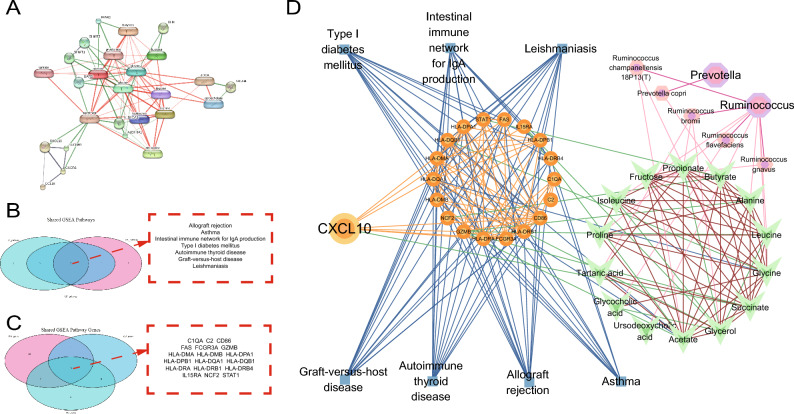
Construction of an interaction network among shared specific gene associated with the gut microbiome in RA, genes related to shared pathways identified via GSEA, and the RA-specific gut microbiome. (**A**) Interaction network of shared specific gene and metabolites associated with the gut microbiome in RA; *CXCL10* was directly associated with the metabolites. (**B**) Venn diagram demonstrating 7 shared high-expression pathways associated with RA and IBD identified via GSEA. (**C**) Venn diagram demonstrating 18 common genes associated with the 7 pathways. (**D**) An interaction network between the, shared specific gene associated with the gut microbiome in RA, shared pathways via GSEA, genes related to shared pathways identified via GSEA, and the RA-specific gut microbiome.

Based on the previous results and data extracted from the gutMGene database, gut microbes associated with RA were identified at the genus (*Ruminococcus*) and species (*Prevotella copri*, *Ruminococcus bromii*, *Ruminococcus flavefaciens*, *Ruminococcus gnavus*, and *Ruminococcus champanellensis 18P13[T]*) levels. The gutMGene database was used to identify metabolites associated with the abovementioned microbes (butyrate, alanine, leucine, isoleucine, glycine, proline, tartaric acid, glycocholic acid, fructose, propionate, glycerol, ursodeoxycholic acid, acetate, and succinate) (Supplementary Spreadsheet S14). Subsequently, the STITCH database was used to identify a single gene (*CXCL10*) directly associated with the metabolites as the shared specific genes related to the gut microbiome in RA (Fig. 8A).

Based on the findings of GSEA and the high expression of *CXCL10* in the RA and IBD samples, Venn diagrams were drawn to demonstrate 7 shared high-expression pathways identified via GSEA and 18 shared genes among these pathways (Fig. 8B,C). Furthermore, an interaction network was established based on the two gut microbial groups identified at the genus level (*Ruminococcus* and *Prevotella*), 5 gut microbial groups identified at the species level (*Prevotella copri*, *Ruminococcus bromii*, *Ruminococcus flavefaciens*, *Ruminococcus gnavus*, and *Ruminococcus champanellensis 18P13[T]*), 14 metabolites (butyrate, alanine, leucine, isoleucine, glycine, proline, tartaric acid, glycocholic acid, fructose, propionate, glycerol, ursodeoxycholic acid, acetate, and succinate) associated with the gut microbiome in RA, and the one shared specific gene related to the gut microbiome (*CXCL10*). This network contained 47 nodes and 231 edges (Fig. 8D). The results suggest that gut microbes associated with RA control the expression of *CXCL10* by altering metabolite content in vivo, thereby regulating the intestinal immune network for IgA synthesis and other pathways.

### Correlation between immune infiltration and shared specific gene related to the gut microbiome in RA

**Figure 9:**
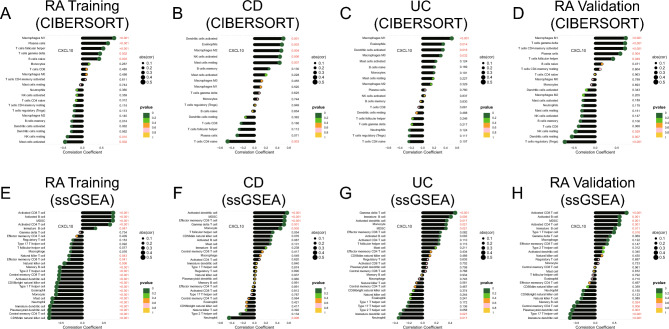
Correlation between immune infiltration and shared specific gene related to the gut microbiome in RA. (**A**) Through the use of the CIBERSORT algorithm, the *CXCL10* expression and immune cells that are entering the body were correlated in the RA training set. (**B**) Through the use of the CIBERSORT algorithm, the *CXCL10* expression and immune cells that are entering the body were correlated in the CD dataset. (**C**) Through the use of the CIBERSORT algorithm, the *CXCL10* expression and immune cells that are entering the body were correlated in the UC dataset. (**D**) Through the use of the CIBERSORT algorithm, the *CXCL10* expression and immune cells that are entering the body were correlated in the RA validation set. (**E**) Through the use of the ssGSEA algorithm, the *CXCL10* expression and immune cells that are entering the body were correlated in the RA training set. (**F**) Through the use of the ssGSEA algorithm, the *CXCL10* expression and immune cells that are entering the body were correlated in the CD dataset. (**G**) Through the use of the ssGSEA algorithm, the *CXCL10* expression and immune cells that are entering the body were correlated in the UC dataset. (**H**) Through the use of the ssGSEA algorithm, the *CXCL10* expression and immune cells that are entering the body were correlated in the RA validation set. The size of the dots indicates the strength of the association between gene expression and immune cell infiltration; the bigger the dots, the greater the correlation. The P-value is represented by the color of the dots; the greener the color, the lower the P-value. Statistical significance was defined as P < 0.05.

In the CIBERSORT algorithm*, CXCL10* had a significant positive correlation with M1 macrophages, plasma cells, follicular helper T cells, naive B cells, and gamma-delta T cells, and a significant negative correlation with resting NK cells and activated Mast cells in the RA training set (Fig. 9A). In the ssGSEA algorithm*, CXCL10* had a significant correlation with activated CD8 T cell, activated B cell, MDSC, activated CD4 T cell, and immature B cell and a significant inverse correlation with CD56dim natural killer cell, central memory CD4 T cell, plasmacytoid dendritic cell, immature dendritic cell, neutrophil, mast cell, monocytein, etc. in the RA training set (Fig. 9E). In the CIBERSORT algorithm*, CXCL10* had a significant positive correlation with activated dendritic cells, eosinophils, M2 macrophages, activated NK cells, and resting mast cells and a significant negative correlation with naïve CD4 T cells (Fig. 9B) in the CD dataset. In the ssGSEA algorithm*, CXCL10* had a significant positive correlation with activated dendritic cells, MDSC, effector memory CD8 T cell, gamma delta T cell, and monocyte and a significant negative correlation with neutrophil in the CD dataset (Fig. 9F). In the CIBERSORT algorithm*, CXCL10* had a significant positive correlation with M0 macrophages, activated dendritic cells, M1 macrophages, and eosinophils in the UC dataset (Fig. 9C). In the ssGSEA algorithm*, CXCL10* had a significant positive correlation with gamma delta T cell, immature B cell, activated dendritic cell, monocyte, and MDSC and a significant negative correlation with immature dendritic cell and neutrophil in the UC dataset (Fig. 9G). In the CIBERSORT algorithm*, CXCL10* had a significant positive correlation with M1 macrophages, gamma-delta T cells, activated memory CD4 T cells, plasma cells, and T follicular helper cells and a significant negative correlation with resting NK cells, resting dendritic cells, and regulatory T cells (Tregs) in the RA validation set (Fig. 9D). In the ssGSEA algorithm, *CXCL10* had a significant positive correlation with activated CD8 T cell, activated B cell, MDSC, activated CD4 T cell, immature B cell, and type 1 T helper cell and a significant negative correlation with immature dendritic cell, type 17 T helper cell, plasmacytoid dendritic cell, central memory CD4 T cell, and memory B cell in the RA validation set (Fig. 9H).

## Discussion

In this study, shared specific genes between IBD and RA were identified via bioinformatic analysis. These genes are associated with the gut microbiome in RA. *CXCL10* was the most relevant gene associated with IBD and RA, which also had a direct relationship with metabolites produced by gut microbes in RA. Additionally, metabolites associated with the gut microbiome in RA and pathways associated with RA and IBD were identified.

*CXCL10* was the most significant shared specific gene between RA and IBD. *CXCL10*, also known as interferon-inducible protein-10 (IP-10), is an ELR-CXC chemokine^54^. It is mostly induced in humans when cell-mediated immune responses are elicited in pathological conditions such as infection, allograft rejection, and autoimmunity^55^. The expression of *CXCL10* is very low in the colonic epithelium but substantially increases in colitis under the induction of IFN-γ^56,57^. Inhibiting *CXCL10* reduces the frequency and severity of colitis and intestinal inflammation^58,59^. Additionally, patients with RA have higher *CXCL10* expression in the synovial membrane^60^. High expression of *CXCL10* mRNA and tissue infiltration of functional proteins in synovial tissue were also reported in CIA model rats with RA in a study on bone marrow mesenchymal stem cell therapy^61^. Increased *CXCL10* mRNA has been observed in liver tissues of patients with HIV and HBV infection in various studies of inflammatory diseases, and its elevation is associated with disease activity^62^. Eldelumab (BMS-936557, formerly known as MDX-1100), a human monoclonal antibody against *CXCL10*, has reportedly been developed and has demonstrated effectiveness in the treatment of RA and IBD^63,64^.

Epidemiological and translational studies have suggested an interaction among bacteria in dysbiotic microbiota, and mucosal locations may play a causative role in the onset of RA^2,65,66^. Considering that the human intestinal microflora is affected by many factors such as region, population, host genetic factors, environment, and diet and different diseases may have different microbial flora, this study aimed to examine the relationship between the intestinal microflora in only one RA dataset and identify shared specific genes between IBD and RA. In the original study of the PRJEB6997 dataset, the abundance of *Lactobacillus salivarius*, *Enterococcus*, and *Bacteroides* was found to be high and that of *Haemophilus*, *Klebsiella*, and *Bifidobacteria* was found to be low in patients with RA^45^. In this study, the abundance of *Prevotella* was high and that of *Ruminococcus* and *Ruminococcus bromii* was low in patients with RA. It’s reported that the abundance of *Prevotella copri* was higher in stool samples of individuals with untreated new-onset RA (between 6 weeks and 6 months after diagnosis), and its presence was associated with a decline in the abundance of *Bacteroides* species and a loss of purportedly beneficial microbes^2^. *Ruminococcus* species can suppress TNF-α, and its abundance is lower in patients with Crohn’s disease than in healthy individuals^67,68^. On the contrary, *Ruminococcus* and *Ruminococcus bromii* are less reported in RA. In this study, data extracted from the gutMGene and STITCH databases revealed some metabolites associated with *Ruminococcus*, and succinate found in *Ruminococcus champanellensis 18P13(T)* had a direct relationship with *CXCL10*. A crucial metabolite in both host and microbial activities is succinate. Although succinate is typically considered an intermediate, it gets accumulated in some pathological conditions, especially during inflammation and metabolic stress^69^. Succinate was considered a pro-inflammatory metabolite; however, a study showed that it exerts anti-inflammatory effects on inflammatory signaling in macrophages^70^.

*CXCL10* was significantly linked with the invasion of M1 macrophages in both RA and UC. Macrophages play an important role in RA. They are commonly found at the cartilage–pannus junction and in inflammatory synovial membranes. The extensive proinflammatory, destructive, and remodeling abilities of macrophages play an important role in both acute and chronic phases of RA^71^. TNF-α and interleukin-1 are two pro-inflammatory cytokines secreted by M1 macrophages, and several experimental and clinical studies have demonstrated their importance in the pathophysiology of RA^72,73^. Additionally, macrophages have been associated with IBD because they play a crucial role in several IBD-related risk genes^74,75^. Macrophages are regionally concentrated and polarize to the M1 subtype in UC, leading to persistent and recurrent inflammation^76–78^.

Although this study had a relatively large sample size (GEO and GMrepo datasets), certain limitations should be noted. Clinical samples should be used to validate these findings. Owing to clinical research and ethical constraints, the present study was not completely rigorous. Moreover, this study focused on the relationship between intestinal microbiota and genes. However, it is challenging to collect or monitor the intestinal microbiota of a single patient at the same time, as the intestinal microbiota may change owing to various factors such as environmental change or growth and development. The use of microarray technology to evaluate gene expression presents another drawback. Since fluorescence-mediated gene expression assessment is a biased method in contrast to hypothesis-free sequencing technology, RNA sequencing is more frequently used for assessing broad gene expression than microarray. Furthermore, even if we attempted to eliminate the batch impact of the combined data using the combat function of the sva package, it is undeniable that any solution can only mitigate this effect. These important issues should be considered in future studies.

In conclusion, because *CXCL10* is involved in the onset of RA and IBD, it can be used to diagnose these two conditions. In addition, the gut microbiome in RA and several pathways related to IBD and RA were also found to be regulated by *CXCL10*. The findings of this study revealed the mechanism underlying the association between RA and IBD and served as a reference for further investigation of the intestinal flora in RA.

## Supplementary Information

Supplementary Information 1.

Supplementary Information 2.

Supplementary Information 3.

Supplementary Information 4.

Supplementary Information 5.

Supplementary Information 6.

Supplementary Information 7.

Supplementary Information 8.

Supplementary Information 9.

Supplementary Information 10.

Supplementary Information 11.

Supplementary Information 12.

Supplementary Information 13.

Supplementary Information 14.

Supplementary Information 15.

## Data Availability

The datasets GSE55235, GSE55457, GSE179285, and GSE77298 for this study can be found in the Gene Expression Omnibus database [https://www.ncbi.nlm.nih.gov/geo/query/acc.cgi?acc=GSE55235/GSE55457/GSE55584/GSE179285/GSE77298/GSE82107]. The RA-related metagenomic sequencing dataset, PRJEB6997, for this study can be found in the GMrepo database [https://gmrepo.humangut.info/data/project/PRJEB6997].
