# Supplementary material for: CXCL10 as a shared specific marker in rheumatoid arthritis and inflammatory bowel disease and a clue involved in the mechanism of intestinal flora in rheumatoid arthritis
Source: Sci Rep. 2023 Jun 16;13:9754. doi: 10.1038/s41598-023-36833-7 (PMC10276029; doi:10.1038/s41598-023-36833-7)
Supplement: Supplementary file 1 — Supplementary Information 1. [file 41598_2023_36833_MOESM1_ESM.docx]

| **Supplement 1. RA_moduleTraitCor** | | |
| --- | --- | --- |
|  | | |
| **Module Color** | **normal** | **RA** |
| **MEturquoise** | **0.0615504** | **-0.0615504** |
| **MEgrey60** | **0.2373534** | **-0.2373534** |
| **MEpink** | **0.3031894** | **-0.3031894** |
| **MEred** | **0.4445004** | **-0.4445004** |
| **MEyellow** | **0.276242** | **-0.276242** |
| **MEdarkgreen** | **0.7404375** | **-0.7404375** |
| **MElightgreen** | **0.1693165** | **-0.1693165** |
| **MEtan** | **0.4687769** | **-0.4687769** |
| **MEdarkgrey** | **-0.1482891** | **0.1482891** |
| **MEmidnightblue** | **-0.4338853** | **0.4338853** |
| **MEpurple** | **-0.3223213** | **0.3223213** |
| **MEwhite** | **-0.2978593** | **0.2978593** |
| **MEblue** | **-0.8240791** | **0.8240791** |
| **MEmagenta** | **-0.6517802** | **0.6517802** |
| **MEsalmon** | **-0.6735658** | **0.6735658** |
| **MEcyan** | **0.2841371** | **-0.2841371** |
| **MEorange** | **-0.2059223** | **0.2059223** |
| **MEroyalblue** | **0.3881285** | **-0.3881285** |
| **MEdarkorange** | **-0.2139736** | **0.2139736** |
| **MEblack** | **0.3545703** | **-0.3545703** |
| **MEgreenyellow** | **0.3468416** | **-0.3468416** |
| **MEdarkturquoise** | **0.5296071** | **-0.5296071** |
| **MElightcyan** | **-0.2473325** | **0.2473325** |
| **MEdarkred** | **0.2851191** | **-0.2851191** |
| **MElightyellow** | **-0.1700159** | **0.1700159** |
| **MEbrown** | **-0.3911705** | **0.3911705** |
| **MEgreen** | **-0.7154272** | **0.7154272** |
| **MEgrey** | **0.2338086** | **-0.2338086** |
