# Supplementary material for: CXCL10 as a shared specific marker in rheumatoid arthritis and inflammatory bowel disease and a clue involved in the mechanism of intestinal flora in rheumatoid arthritis
Source: Sci Rep. 2023 Jun 16;13:9754. doi: 10.1038/s41598-023-36833-7 (PMC10276029; doi:10.1038/s41598-023-36833-7)
Supplement: Supplementary file 2 — Supplementary Information 2. [file 41598_2023_36833_MOESM2_ESM.docx]

| **Supplement 2. RA_moduleTraitPvalue** | | |
| --- | --- | --- |
| **Module Color** | **normal** | **RA** |
| **MEturquoise** | **0.6949901913** | **0.6949901913** |
| **MEgrey60** | **0.12538403468** | **0.12538403468** |
| **MEpink** | **0.04811462722** | **0.04811462722** |
| **MEred** | **0.00282417863** | **0.00282417863** |
| **MEyellow** | **0.0729513634** | **0.0729513634** |
| **MEdarkgreen** | **1.383e-08** | **1.383e-08** |
| **MElightgreen** | **0.27773307954** | **0.27773307954** |
| **MEtan** | **0.00152089526** | **0.00152089526** |
| **MEdarkgrey** | **0.34261859609** | **0.34261859609** |
| **MEmidnightblue** | **0.00365129509** | **0.00365129509** |
| **MEpurple** | **0.0350360668** | **0.0350360668** |
| **MEwhite** | **0.0523887985** | **0.0523887985** |
| **MEblue** | **1e-11** | **1e-11** |
| **MEmagenta** | **2.19806e-06** | **2.19806e-06** |
| **MEsalmon** | **7.4228e-07** | **7.4228e-07** |
| **MEcyan** | **0.06480908555** | **0.06480908555** |
| **MEorange** | **0.18524341456** | **0.18524341456** |
| **MEroyalblue** | **0.01011622998** | **0.01011622998** |
| **MEdarkorange** | **0.16826787469** | **0.16826787469** |
| **MEblack** | **0.01965215155** | **0.01965215155** |
| **MEgreenyellow** | **0.0226897857** | **0.0226897857** |
| **MEdarkturquoise** | **0.00026019885** | **0.00026019885** |
| **MElightcyan** | **0.10981364185** | **0.10981364185** |
| **MEdarkred** | **0.06384896543** | **0.06384896543** |
| **MElightyellow** | **0.27572361599** | **0.27572361599** |
| **MEbrown** | **0.00949344266** | **0.00949344266** |
| **MEgreen** | **6.995e-08** | **6.995e-08** |
| **MEgrey** | **0.13129614971** | **0.13129614971** |
