# Supplementary material for: CXCL10 as a shared specific marker in rheumatoid arthritis and inflammatory bowel disease and a clue involved in the mechanism of intestinal flora in rheumatoid arthritis
Source: Sci Rep. 2023 Jun 16;13:9754. doi: 10.1038/s41598-023-36833-7 (PMC10276029; doi:10.1038/s41598-023-36833-7)
Supplement: Supplementary file 4 — Supplementary Information 4. [file 41598_2023_36833_MOESM4_ESM.docx]

| **Supplement 4. CD_moduleTraitCor** | | |
| --- | --- | --- |
| **Module Color** | **normal** | **CD** |
| **MEplum1** | **-0.2324595** | **0.2324595** |
| **MEwhite** | **-0.2115238** | **0.2115238** |
| **MEbrown** | **-0.0468649** | **0.0468649** |
| **MElightyellow** | **0.1452428** | **-0.1452428** |
| **MEdarkolivegreen** | **-0.2307991** | **0.2307991** |
| **MEbisque4** | **-0.2450851** | **0.2450851** |
| **MEblack** | **-0.2585098** | **0.2585098** |
| **MEmediumpurple3** | **-0.253063** | **0.253063** |
| **MEdarkmagenta** | **-0.6843016** | **0.6843016** |
| **MEroyalblue** | **-0.7381648** | **0.7381648** |
| **MEthistle1** | **0.1350105** | **-0.1350105** |
| **MEtan** | **0.1483264** | **-0.1483264** |
| **MEsteelblue** | **0.2640615** | **-0.2640615** |
| **MEdarkgreen** | **0.1177985** | **-0.1177985** |
| **MEdarkgrey** | **0.1483935** | **-0.1483935** |
| **MEdarkorange** | **0.1426134** | **-0.1426134** |
| **MEbrown4** | **0.1231672** | **-0.1231672** |
| **MEfloralwhite** | **0.172227** | **-0.172227** |
| **MEdarkorange2** | **-0.2180629** | **0.2180629** |
| **MEblue** | **-0.1158069** | **0.1158069** |
| **MEdarkred** | **0.0358891** | **-0.0358891** |
| **MEpaleturquoise** | **0.2503231** | **-0.2503231** |
| **MEyellowgreen** | **0.1330479** | **-0.1330479** |
| **MEdarkslateblue** | **0.439143** | **-0.439143** |
| **MEturquoise** | **0.0037168** | **-0.0037168** |
| **MElightgreen** | **0.2996192** | **-0.2996192** |
| **MEmagenta** | **0.2044941** | **-0.2044941** |
| **MEivory** | **0.1332095** | **-0.1332095** |
| **MEdarkturquoise** | **0.0780829** | **-0.0780829** |
| **MEsienna3** | **-0.0841355** | **0.0841355** |
| **MEsaddlebrown** | **0.0985128** | **-0.0985128** |
| **MEviolet** | **-0.1804565** | **0.1804565** |
| **MElightsteelblue1** | **-0.0454553** | **0.0454553** |
| **MEred** | **-0.2192284** | **0.2192284** |
| **MEgreen** | **-0.063774** | **0.063774** |
| **MEyellow** | **-0.1108084** | **0.1108084** |
| **MEskyblue** | **-0.2259728** | **0.2259728** |
| **MEgrey60** | **-0.2727348** | **0.2727348** |
| **MEplum2** | **-0.2140165** | **0.2140165** |
| **MEpurple** | **-0.1253543** | **0.1253543** |
| **MEorange** | **0.120946** | **-0.120946** |
| **MEpink** | **0.1176498** | **-0.1176498** |
| **MElightcyan1** | **0.0962393** | **-0.0962393** |
| **MEgreenyellow** | **-0.1293911** | **0.1293911** |
| **MElightcyan** | **-0.1966676** | **0.1966676** |
| **MEsalmon** | **0.1250864** | **-0.1250864** |
| **MEmidnightblue** | **0.6115093** | **-0.6115093** |
| **MEorangered4** | **0.1442454** | **-0.1442454** |
| **MEskyblue3** | **0.1528407** | **-0.1528407** |
| **MEcyan** | **0.0861039** | **-0.0861039** |
| **MEthistle2** | **0.1203932** | **-0.1203932** |
| **MEgrey** | **-0.137823** | **0.137823** |
