# Supplementary material for: CXCL10 as a shared specific marker in rheumatoid arthritis and inflammatory bowel disease and a clue involved in the mechanism of intestinal flora in rheumatoid arthritis
Source: Sci Rep. 2023 Jun 16;13:9754. doi: 10.1038/s41598-023-36833-7 (PMC10276029; doi:10.1038/s41598-023-36833-7)
Supplement: Supplementary file 5 — Supplementary Information 5. [file 41598_2023_36833_MOESM5_ESM.docx]

| **Supplement 5. CD_moduleTraitPvalue** | | |
| --- | --- | --- |
|  | | |
| **Module Color** | **normal** | **CD** |
| **MEplum1** | **0.179014** | **0.179014** |
| **MEwhite** | **0.2225389** | **0.2225389** |
| **MEbrown** | **0.7892118** | **0.7892118** |
| **MElightyellow** | **0.4051327** | **0.4051327** |
| **MEdarkolivegreen** | **0.1822271** | **0.1822271** |
| **MEbisque4** | **0.1558894** | **0.1558894** |
| **MEblack** | **0.1337589** | **0.1337589** |
| **MEmediumpurple3** | **0.1424399** | **0.1424399** |
| **MEdarkmagenta** | **5.8e-06** | **5.8e-06** |
| **MEroyalblue** | **4e-07** | **4e-07** |
| **MEthistle1** | **0.4393594** | **0.4393594** |
| **MEtan** | **0.395125** | **0.395125** |
| **MEsteelblue** | **0.1253187** | **0.1253187** |
| **MEdarkgreen** | **0.5003443** | **0.5003443** |
| **MEdarkgrey** | **0.3949086** | **0.3949086** |
| **MEdarkorange** | **0.4137794** | **0.4137794** |
| **MEbrown4** | **0.4808756** | **0.4808756** |
| **MEfloralwhite** | **0.3225052** | **0.3225052** |
| **MEdarkorange2** | **0.2082344** | **0.2082344** |
| **MEblue** | **0.5076666** | **0.5076666** |
| **MEdarkred** | **0.837824** | **0.837824** |
| **MEpaleturquoise** | **0.1469596** | **0.1469596** |
| **MEyellowgreen** | **0.4461002** | **0.4461002** |
| **MEdarkslateblue** | **0.0083092** | **0.0083092** |
| **MEturquoise** | **0.983094** | **0.983094** |
| **MElightgreen** | **0.0803567** | **0.0803567** |
| **MEmagenta** | **0.2386493** | **0.2386493** |
| **MEivory** | **0.445543** | **0.445543** |
| **MEdarkturquoise** | **0.6557055** | **0.6557055** |
| **MEsienna3** | **0.6308515** | **0.6308515** |
| **MEsaddlebrown** | **0.5734241** | **0.5734241** |
| **MEviolet** | **0.2995634** | **0.2995634** |
| **MElightsteelblue1** | **0.7954156** | **0.7954156** |
| **MEred** | **0.2057533** | **0.2057533** |
| **MEgreen** | **0.7158875** | **0.7158875** |
| **MEyellow** | **0.5262767** | **0.5262767** |
| **MEskyblue** | **0.1917972** | **0.1917972** |
| **MEgrey60** | **0.1129344** | **0.1129344** |
| **MEplum2** | **0.2170091** | **0.2170091** |
| **MEpurple** | **0.473059** | **0.473059** |
| **MEorange** | **0.4888825** | **0.4888825** |
| **MEpink** | **0.5008892** | **0.5008892** |
| **MElightcyan1** | **0.582346** | **0.582346** |
| **MEgreenyellow** | **0.4588085** | **0.4588085** |
| **MElightcyan** | **0.2574882** | **0.2574882** |
| **MEsalmon** | **0.474013** | **0.474013** |
| **MEmidnightblue** | **9.51e-05** | **9.51e-05** |
| **MEorangered4** | **0.4084004** | **0.4084004** |
| **MEskyblue3** | **0.3807336** | **0.3807336** |
| **MEcyan** | **0.6228519** | **0.6228519** |
| **MEthistle2** | **0.4908856** | **0.4908856** |
| **MEgrey** | **0.4297974** | **0.4297974** |
