# Supplementary material for: CXCL10 as a shared specific marker in rheumatoid arthritis and inflammatory bowel disease and a clue involved in the mechanism of intestinal flora in rheumatoid arthritis
Source: Sci Rep. 2023 Jun 16;13:9754. doi: 10.1038/s41598-023-36833-7 (PMC10276029; doi:10.1038/s41598-023-36833-7)
Supplement: Supplementary file 7 — Supplementary Information 7. [file 41598_2023_36833_MOESM7_ESM.docx]

| **Supplement 7. UC_moduleTraitCor** | | |
| --- | --- | --- |
| **Module Color** | **normal** | **UC** |
| **MEgreenyellow** | **-0.0399648** | **0.0399648** |
| **MElightcyan** | **0.1698869** | **-0.1698869** |
| **MElightgreen** | **-0.1480705** | **0.1480705** |
| **MEpink** | **-0.1792958** | **0.1792958** |
| **MEplum1** | **-0.1675825** | **0.1675825** |
| **MEmagenta** | **-0.1695694** | **0.1695694** |
| **MEgrey60** | **-0.1733898** | **0.1733898** |
| **MEyellowgreen** | **-0.778319** | **0.778319** |
| **MEdarkgrey** | **-0.3249042** | **0.3249042** |
| **MElightsteelblue1** | **-0.5477976** | **0.5477976** |
| **MEroyalblue** | **-0.105868** | **0.105868** |
| **MEivory** | **-0.1512275** | **0.1512275** |
| **MEsalmon** | **-0.1662401** | **0.1662401** |
| **MEcyan** | **-0.2596089** | **0.2596089** |
| **MEviolet** | **-0.1086981** | **0.1086981** |
| **MEdarkgreen** | **-0.2880262** | **0.2880262** |
| **MEbrown** | **-0.4391721** | **0.4391721** |
| **MEmediumpurple3** | **-0.4778256** | **0.4778256** |
| **MEdarkorange** | **0.1713338** | **-0.1713338** |
| **MEblack** | **-0.1542218** | **0.1542218** |
| **MEyellow** | **0.1830656** | **-0.1830656** |
| **MEsalmon4** | **-0.1880945** | **0.1880945** |
| **MEwhite** | **-0.1653819** | **0.1653819** |
| **MEorange** | **0.188427** | **-0.188427** |
| **MEpaleturquoise** | **0.1534645** | **-0.1534645** |
| **MEsaddlebrown** | **0.1642065** | **-0.1642065** |
| **MEdarkslateblue** | **0.130536** | **-0.130536** |
| **MEdarkorange2** | **0.1747588** | **-0.1747588** |
| **MEsteelblue** | **0.1293432** | **-0.1293432** |
| **MEbisque4** | **0.1606998** | **-0.1606998** |
| **MEpurple** | **-0.1350215** | **0.1350215** |
| **MEorangered4** | **0.1640139** | **-0.1640139** |
| **MElightyellow** | **0.4924179** | **-0.4924179** |
| **MEskyblue** | **0.4897505** | **-0.4897505** |
| **MEsienna3** | **0.1305077** | **-0.1305077** |
| **MEtan** | **0.5020021** | **-0.5020021** |
| **MElightcyan1** | **0.4532428** | **-0.4532428** |
| **MEdarkmagenta** | **0.3352508** | **-0.3352508** |
| **MEskyblue3** | **0.5047164** | **-0.5047164** |
| **MEthistle2** | **0.2364064** | **-0.2364064** |
| **MEdarkred** | **0.1498788** | **-0.1498788** |
| **MEred** | **0.1192436** | **-0.1192436** |
| **MEdarkturquoise** | **0.1407016** | **-0.1407016** |
| **MEfloralwhite** | **0.141615** | **-0.141615** |
| **MEdarkolivegreen** | **-0.143283** | **0.143283** |
| **MEmidnightblue** | **-0.0326109** | **0.0326109** |
| **MEnavajowhite2** | **-0.1766776** | **0.1766776** |
| **MEmaroon** | **-0.146511** | **0.146511** |
| **MElightpink4** | **0.1277251** | **-0.1277251** |
| **MEbrown4** | **-0.1252514** | **0.1252514** |
| **MEpalevioletred3** | **-0.1648791** | **0.1648791** |
| **MEthistle1** | **-0.1610563** | **0.1610563** |
| **MEplum2** | **-0.1487187** | **0.1487187** |
| **MEturquoise** | **0.0571864** | **-0.0571864** |
| **MEblue** | **0.3718231** | **-0.3718231** |
| **MEgreen** | **0.1236761** | **-0.1236761** |
| **MEgrey** | **0.0763099** | **-0.0763099** |
