# Supplementary material for: CXCL10 as a shared specific marker in rheumatoid arthritis and inflammatory bowel disease and a clue involved in the mechanism of intestinal flora in rheumatoid arthritis
Source: Sci Rep. 2023 Jun 16;13:9754. doi: 10.1038/s41598-023-36833-7 (PMC10276029; doi:10.1038/s41598-023-36833-7)
Supplement: Supplementary file 8 — Supplementary Information 8. [file 41598_2023_36833_MOESM8_ESM.docx]

| **Supplement 8. UC_moduleTraitPvalue** | | |
| --- | --- | --- |
| **Module Color** | **normal** | **UC** |
| **MEgreenyellow** | **0.7943608493** | **0.7943608493** |
| **MElightcyan** | **0.2645504189** | **0.2645504189** |
| **MElightgreen** | **0.3316972927** | **0.3316972927** |
| **MEpink** | **0.2386028994** | **0.2386028994** |
| **MEplum1** | **0.2711810589** | **0.2711810589** |
| **MEmagenta** | **0.2654575872** | **0.2654575872** |
| **MEgrey60** | **0.2546795313** | **0.2546795313** |
| **MEyellowgreen** | **3e-10** | **3e-10** |
| **MEdarkgrey** | **0.0294319477** | **0.0294319477** |
| **MElightsteelblue1** | **9.82509e-05** | **9.82509e-05** |
| **MEroyalblue** | **0.488844049** | **0.488844049** |
| **MEivory** | **0.3213753303** | **0.3213753303** |
| **MEsalmon** | **0.2750935138** | **0.2750935138** |
| **MEcyan** | **0.0850389438** | **0.0850389438** |
| **MEviolet** | **0.4772326622** | **0.4772326622** |
| **MEdarkgreen** | **0.0550291628** | **0.0550291628** |
| **MEbrown** | **0.0025433383** | **0.0025433383** |
| **MEmediumpurple3** | **0.0009019311** | **0.0009019311** |
| **MEdarkorange** | **0.2604428988** | **0.2604428988** |
| **MEblack** | **0.3117739551** | **0.3117739551** |
| **MEyellow** | **0.2287100493** | **0.2287100493** |
| **MEsalmon4** | **0.2159573427** | **0.2159573427** |
| **MEwhite** | **0.2776143772** | **0.2776143772** |
| **MEorange** | **0.2151320566** | **0.2151320566** |
| **MEpaleturquoise** | **0.3141849299** | **0.3141849299** |
| **MEsaddlebrown** | **0.2810915448** | **0.2810915448** |
| **MEdarkslateblue** | **0.3927246943** | **0.3927246943** |
| **MEdarkorange2** | **0.2508898746** | **0.2508898746** |
| **MEsteelblue** | **0.3971010379** | **0.3971010379** |
| **MEbisque4** | **0.2916336728** | **0.2916336728** |
| **MEpurple** | **0.3765218142** | **0.3765218142** |
| **MEorangered4** | **0.2816640053** | **0.2816640053** |
| **MElightyellow** | **0.0005903243** | **0.0005903243** |
| **MEskyblue** | **0.0006387806** | **0.0006387806** |
| **MEsienna3** | **0.3928284424** | **0.3928284424** |
| **MEtan** | **0.0004422441** | **0.0004422441** |
| **MElightcyan1** | **0.0017681614** | **0.0017681614** |
| **MEdarkmagenta** | **0.024370651** | **0.024370651** |
| **MEskyblue3** | **0.0004068814** | **0.0004068814** |
| **MEthistle2** | **0.1179374304** | **0.1179374304** |
| **MEdarkred** | **0.3257597852** | **0.3257597852** |
| **MEred** | **0.4352785789** | **0.4352785789** |
| **MEdarkturquoise** | **0.3565849606** | **0.3565849606** |
| **MEfloralwhite** | **0.3534398475** | **0.3534398475** |
| **MEdarkolivegreen** | **0.347740465** | **0.347740465** |
| **MEmidnightblue** | **0.8315914031** | **0.8315914031** |
| **MEnavajowhite2** | **0.2456422848** | **0.2456422848** |
| **MEmaroon** | **0.3368719328** | **0.3368719328** |
| **MElightpink4** | **0.4030831209** | **0.4030831209** |
| **MEbrown4** | **0.4123277565** | **0.4123277565** |
| **MEpalevioletred3** | **0.2790984318** | **0.2790984318** |
| **MEthistle1** | **0.2905505194** | **0.2905505194** |
| **MEplum2** | **0.3295614517** | **0.3295614517** |
| **MEturquoise** | **0.7090525952** | **0.7090525952** |
| **MEblue** | **0.0119043571** | **0.0119043571** |
| **MEgreen** | **0.4182778164** | **0.4182778164** |
| **MEgrey** | **0.6183253576** | **0.6183253576** |
