# Supplementary material for: CXCL10 as a shared specific marker in rheumatoid arthritis and inflammatory bowel disease and a clue involved in the mechanism of intestinal flora in rheumatoid arthritis
Source: Sci Rep. 2023 Jun 16;13:9754. doi: 10.1038/s41598-023-36833-7 (PMC10276029; doi:10.1038/s41598-023-36833-7)
Supplement: Supplementary file 15 — Supplementary Information 15. [file 41598_2023_36833_MOESM15_ESM.pdf]

**Title:Shared Specific Markers in Rheumatoid Arthritis and Inflammatory Bowel Disease: Clues to the Mechanism of Intestinal Flora in Rheumatoid Arthritis**

Author list:

Yin GUAN[1], Yue Zhang[1], Yifan Zhu[1], Yue WANG[2]*

[1]Affiliated Hospital of Nanjing University of Chinese Medicine, Nanjing, Jiangsu, 210029.

[2]Department of Rheumatism Immunity Branch, Affiliated Hospital of Nanjing University of Chinese Medicine, Nanjing, Jiangsu, 210029.

***Corresponding author:** Yue WANG, Department of Rheumatism Immunity Branch, Affiliated Hospital of Nanjing University of Chinese Medicine, No. 155 Hanzhong Road, Qinhuai, Nanjing, Jiangsu, 210029.

Supplement 1. RA_moduleTraitCor
Supplement 2. RA_moduleTraitPvalue
Supplement 3. RA_WGCNA_module genes
Supplement 4. CD_moduleTraitCor
Supplement 5. CD_moduleTraitPvalue
Supplement 6. CD_WGCNA_module genes
Supplement 7. UC_moduleTraitCor
Supplement 8. UC_moduleTraitPvalue
Supplement 9. UC_WGCNA_module genes
Supplement 10. Degree Rank_RA
Supplement 11. Degree Rank_CD
Supplement 12. Degree Rank_UC
Supplement 13. Differential analysis of the intestinal flora in rheumatoid arthritis
Supplement 14. Rheumatoid arthritis gut microbiota and metabolites
